# Community engagement group model in basic and biomedical research: lessons learned from the BEAT-HIV Delaney Collaboratory towards an HIV-1 cure

**DOI:** 10.1186/s40900-023-00449-y

**Published:** 2023-06-08

**Authors:** Karine Dubé, Beth Peterson, Nora L. Jones, Amy Onorato, William B. Carter, Christine Dannaway, Steven Johnson, Roy Hayes, Marcus Hill, Rease Maddox, James L. Riley, Jane Shull, David Metzger, Luis J. Montaner

**Affiliations:** 1 Division of Infectious Diseases and Global Public Health, School of Medicine, University of California San Diego, San Diego, CA USA; 2 Health Policy and Management, UNC Gillings School of Global Public Health, Chapel Hill, NC USA; 3 Wistar Institute, 3601 Spruce Street, Room 480, Philadelphia, PA 19104 USA; 4 BEAT-HIV Delaney Collaboratory Community Advisory Board (CAB), Philadelphia, PA USA; 5 Department of Psychiatry, University of Pennsylvania, Philadelphia, PA USA; 6 Department of Microbiology, Perelman School of Medicine, University of Pennsylvania, Philadelphia, PA USA; 7 Philadelphia FIGHT Community Health Centers, Philadelphia, PA USA

**Keywords:** Community engagement, Patient and public involvement, Community advisory board, Community-based organizations, Advocacy, HIV cure research, Lessons learned

## Abstract

**Introduction:**

Achieving effective community engagement has been an objective of U.S. National Institutes of Health-funded HIV research efforts, including participation of persons with HIV. Community Advisory Boards (CABs) have remained the predominant model for community engagement since their creation in 1989. As HIV cure-directed research efforts have grown into larger academic-industry partnerships directing resources toward both basic and clinical research under the Martin Delaney Collaboratories (MDC), community input models have also evolved. The BEAT-HIV MDC Collaboratory, based at The Wistar Institute in Philadelphia, United States, implemented a three-part model for community engagement that has shown success in providing greater impact for community engagement across basic, biomedical, and social sciences research efforts.

**Discussion:**

In this paper, we review the case study of the formation of the BEAT-HIV Community Engagement Group (CEG) model, starting with the historical partnership between The Wistar Institute as a basic research center and Philadelphia FIGHT as a not-for-profit community-based organization (CBO), and culminating with the growth of community engagement under the BEAT-HIV MDC. Second, we present the impact of a cooperative structure including a Community Advisory Board (CAB), CBO, and researchers through the BEAT-HIV CEG model, and highlight collaborative projects that demonstrate the potential strengths, challenges, and opportunities of this model. We also describe challenges and future opportunities for the use of the CEG model.

**Conclusions:**

Our CEG model integrating a CBO, CAB and scientists could help move us towards the goal of effective, equitable and ethical engagement in HIV cure-directed research. In sharing our lessons learned, challenges and growing pains, we contribute to the science of community engagement into biomedical research efforts with an emphasis on HIV cure-directed research. Our documented experience with implementing the CEG supports greater discussion and independent implementation efforts for this model to engage communities into working teams in a way we find a meaningful, ethical, and sustainable model in support of basic, clinical/biomedical, social sciences and ethics research.

**Supplementary Information:**

The online version contains supplementary material available at 10.1186/s40900-023-00449-y.

## Abstract (plain language summary)

HIV biomedical research groups have prioritized community support and representation as exemplified by the creation of Community Advisory Boards (CABs). Most CABs bring diverse stakeholders to advise on research objectives as part of their activities. The BEAT-HIV Delaney Collaboratory, based at The Wistar Institute in Philadelphia, is a research program created in 2016 to advance HIV cure research. To better engage communities beyond the CAB, the BEAT-HIV Delaney Collaboratory created a Community Engagement Group (CEG) model composed of three distinct components. First, the involvement of a community-based organization (CBO) introduces the historical know-how and relationship with the community. Philadelphia FIGHT fulfills the CBO role as a provider of primary care, education, advocacy, and research support for persons with HIV. Second, the BEAT-HIV CAB provides individual experiences and community input into HIV cure research and gives updates to the broader community about the status of research. Third, basic, clinical/biomedical, and social scientists implement the scientific goals of the BEAT-HIV Collaboratory. In this paper, we aimed to highlight the strengths, challenges, lessons learned, and opportunities of the BEAT-HIV CEG model. We also present examples of collaborative community engagement projects. Our paper contributes to the literature on novel community engagement approaches beyond the CAB. Based on our experience to date using the CEG, a multi-part community engagement model could help move us towards the goal of inclusive, effective, equitable, and ethical engagement in HIV cure research.

**Supplementary Information:**

The online version contains supplementary material available at 10.1186/s40900-023-00449-y.

## Background

Tenets of meaningful patient and community involvement in HIV research were first articulated in the 1983 Denver Principles [1] and later endorsed by the Joint United Nations Programme on HIV/AIDS (UNAIDS) as the principles of Greater Involvement of People Living with HIV (GIPA) [2]. Although there is no consensus statement determining when patient and community involvement officially becomes “meaningful,” the GIPA principles aim to achieve people with HIV (PWH)’s rights to self-determination and participation in decision-making processes that affect their lives. Consequently, the rapid advancements in HIV treatment and prevention options since the early 1980s were propelled by the increasingly organized engagement of communities most affected by HIV [3, 4]. Yet pathways toward meaningful engagement in HIV research have been marked with inequities and ethical conflicts, as evidenced, for example, by the closure of HIV pre-exposure prophylaxis (PrEP) trial sites in Cameroon and Cambodia in 2004 after failure to meaningfully engage affected communities [4]. Following these premature trial closures, UNAIDS, the World Health Organization (WHO) and AVAC developed Good Participatory Practice (GPP) guidelines [5] designed to improve biomedical researchers’ engagement of communities in trial design and implementation. GPP relies on six principles of engagement that include respect, mutual understanding, integrity, transparency, accountability, and community stakeholder autonomy [5]. GPP principles have since been adapted to biomedical research on tuberculosis [6], emerging pathogens [7] and COVID-19 [8].

In 1997, the U.S. Centers for Disease Control and Prevention (CDC) defined community engagement as “the process of working collaboratively with and through groups of people affiliated by geographic proximity, special interest, or similar situations to address issues affecting the well-being of those people [9].” In this paper, we used the 1997 CDC community engagement definition to guide our discussions of patient and public engagement (PPI). Further, Dickert and Sugarman [10] put forth four ethical goals of community engagement, namely: enhanced protection of the patient population, enhanced benefits, legitimacy of the research, and shared responsibility. To meet these ethical responsibilities, researchers must engage communities through different phases of the research process, from defining the research questions, designing the research, interpreting findings, to sharing data with engaged communities [11].

In HIV research efforts, the creation of Community Advisory Boards (CABs) has become the main strategy for researchers to introduce community representation and input in the HIV research process. CABs have their roots in the late 1980s when PWH demanded greater involvement in HIV research efforts. The first CABs were created through the National Institute of Allergy and Infectious Diseases (NIAID) in 1989, and since then have become a requirement for federally funded HIV research trial networks and their participating clinical trial sites [12, 13]. CABs are charged with providing input into the research process, acting as a sounding board, engaging in participatory research, and advancing community education. The composition of CABs seeks to include persons with diverse backgrounds with respect to racial and ethnic identity, sex and gender identification, age, geography, skills and cultural background—among other characteristics—to allow the provision of broad-based input on HIV research efforts. While election of CAB members facilitates bringing a variety of community members together for advice and input, CABs are sometimes not independent working units in the community aside from the project they are linked to (i.e., CABs can be dissolved when projects end, and participation is oftentimes voluntary). This fact may reflect a structural and ethical limitation on the ongoing impact of stable CAB community voices that has proven critical to HIV research efforts as well as in identifying future research needs. Conversely, however, frequent CAB member turnover can also help reflect changes in patient or participant populations of interest, and may provide for a perspective not otherwise held by long-term CAB members.

In 2011, the U.S. National Institutes of Health (NIH) launched a research initiative called the Martin Delaney Collaboratories (MDCs) for HIV Cure Research, funded in five-year cycles [14]. Named in honor of the late community activist Martin Delaney, the aim of the MDCs is to foster multidisciplinary collaborations to study HIV persistence and develop potential HIV cure-directed research strategies [14]. These strategies could include approaches to either eliminate HIV from the body or keep HIV durably suppressed in the absence of antiretroviral treatment (ART) (hereafter referred to as “cure-directed research”). The Beyond Antiretroviral Therapy - HIV (BEAT-HIV) Collaboratory, based at The Wistar Institute in Philadelphia, United States, was created in 2016 and refunded in 2021 as one of ten MDCs taking part in the third funding cycle (2021–2026).

**Fig. 1:**
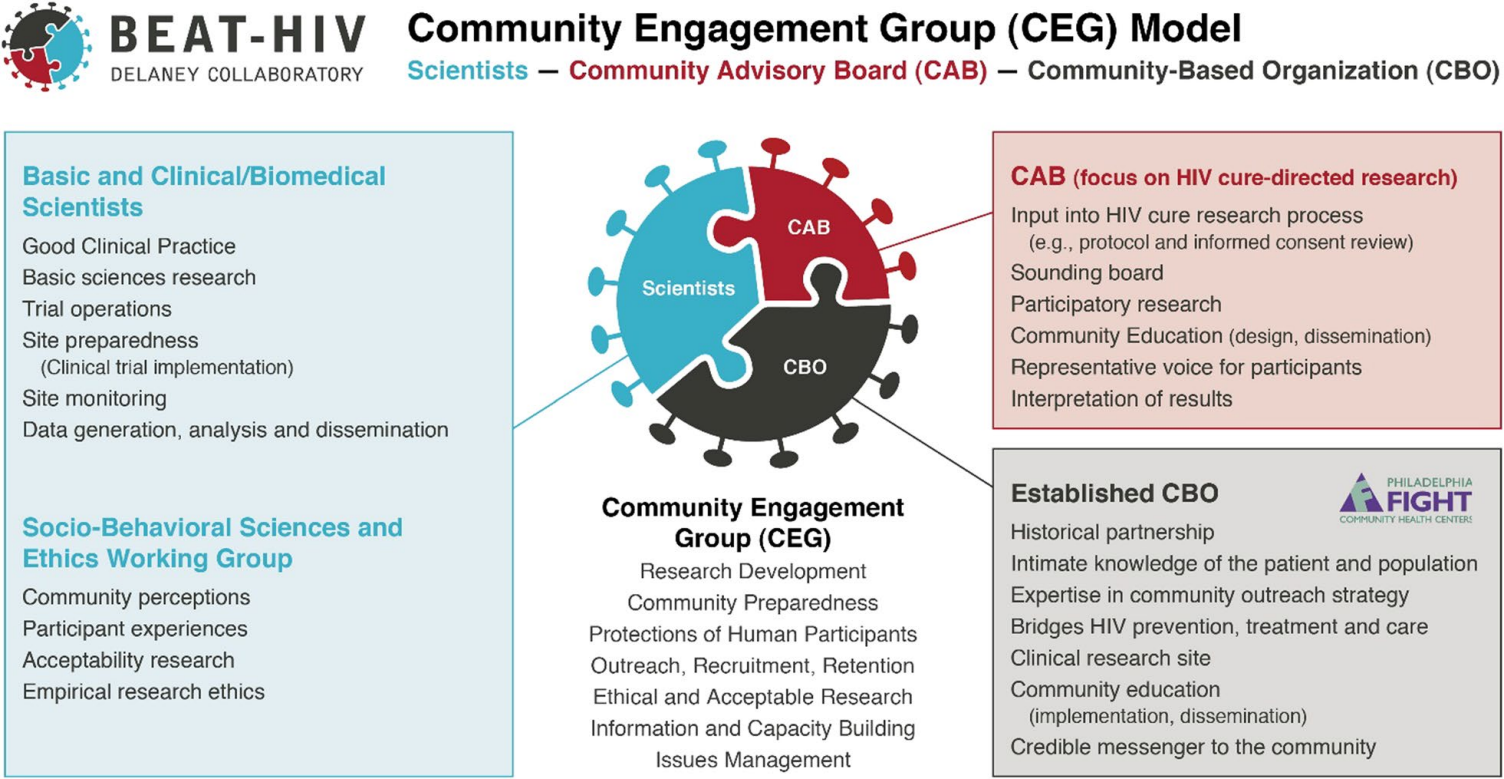
BEAT-HIV Martin Delaney Collaboratory community engagement group (CEG) model

In this paper, we present a case study of how the BEAT-HIV Collaboratory addressed the need for structurally sustainable relationships with community voices through the creation of a community engagement group (CEG) model. The CEG is a tripartite model for meaningful community engagement in HIV cure-directed research linking basic, clinical/biomedical, and social scientists together with a community-based organization (CBO)—Philadelphia FIGHT—and an independent CAB (Fig. 1). First, we review the formation of the BEAT-HIV CEG, starting with the historical partnership between The Wistar Institute and Philadelphia FIGHT. Second, we present the impact of introducing a CAB through the BEAT-HIV CEG model and highlight collaborative working groups and projects that demonstrate the potential strengths, challenges, and opportunities of this model. We then describe some of our practical lessons learned to date using the CEG model. Collective reflections presented in this manuscript were informed by meetings with BEAT-HIV-affiliated community members who participated in discussions regarding potential strengths, challenges and lessons learned about the CEG model, and who helped shape and review the contents of this manuscript over its multiple iterations. Our paper is a direct response to the limited documentation of lessons learned and potential best practices in the field of community engagement in HIV cure-directed research. The present case study on the CEG model is intended to contribute to the growing literature on community engagement in HIV cure-directed research.

## Discussion

### Evolution and presentation of the BEAT-HIV CEG model

The BEAT-HIV CEG model was created by the BEAT-HIV Collaboratory leadership and brings together three independent sets of partners: 1) an established CBO providing health and social services to PWH (Philadelphia FIGHT, fight.org), 2) a cure-directed CAB, and 3) basic, clinical/biomedical, and social scientists (beat-hiv.org). The CEG model is informed by principles of health equity, social justice, reciprocity, participation of underrepresented groups, and integration. Table 1 summarizes principles for participating groups in the CEG model.

**Table 1:** Principles for participating groups in a CEG framework

Sharing a common vision to integrate efforts
Democratic group control on agenda and projects
Independent contributions to group programmatic goals
Financial autonomy between groups
Shared leadership assignments
Maintain independence while strengthening collaboration

The CEG model was conceptualized and implemented at the inception of the BEAT-HIV Collaboratory in 2016 (see below) and has continued to evolve. Table 2 provides a summary timeline of BEAT-HIV CEG-relevant events presented in this manuscript.

**Table 2:** Summary timeline of key BEAT-HIV community engagement group events

*1989:* Community Advisory Boards became a requirement for federally funded HIV research trial networks and their participating clinical trial sites
*1990:* Philadelphia FIGHT is established in Philadelphia, PA
*1995:* Collaboration between Philadelphia FIGHT and Wistar Institute begins
*2011:* U.S. National Institutes of Health starts Martin Delaney Collaboratory program towards an HIV-1 cure
*2013:* U.S. National Institutes of Health mandates inclusion of community engagement in Martin Delaney Collaboratory-funded HIV cure research efforts
*2016:* BEAT-HIV Delaney Collaboratory focused on basic and clinical/biomedical objectives is created (funded in 2016–2021 cycle)
*2016:* BEAT-HIV Community Advisory Board is created at the Wistar Institute, Philadelphia, PA
*2019:* BEAT-HIV cure-directed research education video series premieres in Philadelphia (*The Philadelphia Story*)
*2019:* BEAT-HIV Delaney Collaboratory adds Social Sciences and Ethics Working Group
*2019:* BEAT-HIV implements Community Advisory Board-led Survey of HIV Clinic Patients in Philadelphia, PA
*2019–2022:* BEAT-HIV implements investigator-led home-based viral load acceptability study
*2019–2023:* BEAT-HIV implements Community Advisory Board-led Participant Experience Study (social sciences project)
*2020:* BEAT-HIV Community Engagement Group publishes position paper on analytical treatment interruptions
*2020:* U.S. National Institutes of Health mandates inclusion of community engagement coordinator in addition to CAB in new cycle of Martin Delaney Collaboratory-funded HIV cure research efforts
*2021:* BEAT-HIV Delaney Collaboratory is renewed (funded in 2021–2026 cycle)

The CEG model provides a platform for acceptable and ethical research development, community preparedness, protection of human participants, outreach, recruitment and retention of participants, information and capacity building, and issues management. To be sustainable, the CEG model requires ongoing shared conversations, dynamic representation, and equitable financial support for each of the three components of the tripartite structure. The CEG requires financial resources directed to all three parts, time commitments to maintain relationships, mutual (occasionally contentious) dialogues, and ongoing tri-directional capacity building of CEG members. Specifically, the CEG is supported by 5.25% of direct funds available to the BEAT-HIV MDC allocated in equal 1.75% subcontracts to support the CAB structure (subcontract with University of Pennsylvania), Philadelphia FIGHT outreach efforts (subcontract with Philadelphia FIGHT), and social sciences research (subcontract with host institution for lead social sciences researcher). The BEAT-HIV leadership holds each of these “subcontracts” accountable under a yearly unified CEG progress report upon renewal of future year budgets. Day-to-day oversight of CAB and larger CEG operations are handled by a community engagement coordinator (correspondence, meeting set-up, committees) based at the University of Pennsylvania with added support from the BEAT-HIV administration effort. All CAB costs are supported by its dedicated subcontract including provision of compensation for CAB members intended to reimburse travel and parking expenses. As of 2023, we provide $30 for each CEG meeting attended with an extra $30 per committee meeting attendance ($60 maximum per month). Amounts provided were established by the BEAT-HIV leadership and remain an active area of discussion with CAB members based on the added demands on time that arise for CEG projects and services (see Challenges section below).

The larger agenda for the CEG is based on priorities defined to accomplish community engagement goals described in the proposal. A yearly CEG retreat focused on strategic planning also supports defining major action items for the group. A month-to-month agenda for meetings is set by the CAB officers in consultation with BEAT-HIV Principal Investigators. Administrative support for day-to-day oversight of CAB, CAB or CEG committees, and larger CEG operations are handled by a community engagement coordinator (correspondence, meeting set-up, committees) based at the University of Pennsylvania with added support from the BEAT-HIV administration effort. CAB/CEG meeting agenda (see generalized example of CAB agenda in Additional file 1: Appendix 1) is chaired by the CAB Chair and include discussions and CAB-only voting items (i.e., voting in new CAB/CEG members) as well as CEG project review where all members vote and participate. All CEG groups (CAB, CBO, Social Scientists and BEAT-HIV Principal Investigators) are included in each monthly agenda ensuring their input into existing or new project directions. Working group committees are determined at CAB/CEG meetings.

#### Established community-based organization (CBO)

Philadelphia FIGHT Community Health Centers is an HIV/AIDS service organization based in Philadelphia that provides primary care, education, advocacy, and research. The organization was formed in 1990 as a partnership of PWH and HIV clinicians, using social and racial justice approaches to HIV prevention, treatment, and care. In 1995, Philadelphia FIGHT started a collaboration with Dr. Luis Montaner and The Wistar Institute based on a shared vision to advance research toward an HIV cure, and to educate PWH on the importance of basic research. Notably, Philadelphia FIGHT’s Executive Director, Jane Shull, oversaw the expansion of the organization from a small start-up non-governmental organization (NGO) to a Federally Qualified Health Center (FQHC) serving underserved communities in the Greater Philadelphia area. When the BEAT-HIV Collaboratory was formed in 2016 with a larger number of local and out-of-state researchers, expanding the existing relationship with Philadelphia FIGHT represented a natural extension of community engagement efforts already in place with The Wistar Institute. Therefore, the creation of the CEG model, adding a CAB as a third interdependent component in the BEAT-HIV Collaboratory, rested on a 25-year foundational partnership engaging clients, basic researchers and clinicians in HIV-related research. Several of the Philadelphia community members who would subsequently be engaged to serve on the newly formed BEAT-HIV CAB had prior interactions with Philadelphia FIGHT as either clients, trainees, staff, or as study participants in The Wistar Institute’s research. Building on these prior connections fostered trust between all components of the CEG and supported early efforts to engage, inform, and advocate for PWH and affected communities in HIV cure-directed research.

In the BEAT-HIV CEG model, Philadelphia FIGHT serves as the established CBO, providing intimate knowledge of the patient or participant population, established communication platforms, expertise in community outreach (including the use of social media), and credible messaging from and to the community. The role of Philadelphia FIGHT as a partner with The Wistar Institute has been supported by funding from the start and through the BEAT-HIV NIH awards with independent subcontracts for 1.75% of total direct fund award (equal to the CAB budget). Philadelphia FIGHT is therefore an equal research and institutional partner with regard to project budget planning and voting representation at CEG meetings, countering the strongly held view that academic efforts “take” more than they “give” when approaching non-academic partners.

Philadelphia FIGHT guides the implementation of the BEAT-HIV CEG local community engagement plan while providing programmatic support for the CEG for community education and outreach efforts. The specific activities supported at Philadelphia FIGHT reflect the community engagement portion of proposal at onset and ongoing priorities of the CEG each year. For example, Philadelphia FIGHT organizes an annual AIDS Education Month and HIV Prevention Summit which provides a platform for BEAT-HIV’s data dissemination activities with a broad base of diverse community constituents (with respect to race, ethnicity, gender, sex, and age). Philadelphia FIGHT also allows BEAT-HIV researchers to bridge HIV cure-directed research with available HIV prevention, treatment, and care services in the community to permit a more comprehensive approach to HIV cure-directed research.

As a result of the partnership, Philadelphia FIGHT’s HIV clinicians provide a critical role in reviewing and/or endorsing HIV cure-directed trials implemented by or in collaboration with the BEAT-HIV Collaboratory. Philadelphia FIGHT is also a well-established clinical research site implementing their own clinical trials aligned with the BEAT-HIV’s scientific objectives. Philadelphia FIGHT clinicians refer PWH to available HIV cure-directed clinical trials based on trusted doctor/researcher—patient/participant relationships; however, there remains a careful delineation of responsibilities between provision of HIV care and HIV clinical research.

#### Community advisory board (CAB)

The BEAT-HIV CAB was established in 2016. BEAT-HIV CAB members are focused on providing community input into HIV cure-directed research efforts and informing the broader community about the status of research. In forming the BEAT-HIV CAB, the Collaboratory leadership was mindful to acknowledge core elements of community as proposed by MacQueen and colleagues: locus (sense of place), sharing, joint action, social ties, and diversity [15]. BEAT-HIV CAB members have represented a range of constituencies, skills and lived experiences. CAB members provide feedback on various aspects of research (including, for example, reviewing research protocols, informed consent forms, and participant materials), disseminate research findings in lay terms to communities of interest, and relay suggestions from the community to the BEAT-HIV leadership. BEAT-HIV CAB operating procedures (also called “bylaws” by CAB members) describe procedures for nomination and approval of members and management of CAB-related activities.

The BEAT-HIV CAB meetings are embedded within monthly CEG meetings, and ad hoc sub-committee or working group meetings occur as necessary. CEG/CAB meetings are chaired by the CAB Chair. The CAB meetings provide a forum for all CEG components to interact, providing updates on activities and planning for future events. The CAB also has the option to host added CAB-only meetings as needed. An experienced community engagement coordinator based at the University of Pennsylvania provides programmatic support for all CAB-related activities (e.g., coordinating meetings, including drafting meeting agendas in collaboration with CAB members, etc.). The CAB receives separate funding to conduct its activities under a subcontract to the University of Pennsylvania with 1.75% of total direct costs of the BEAT-HIV program.

#### BEAT-HIV collaboratory scientists

Basic, clinical, social, and biomedical scientists of the BEAT-HIV Collaboratory implement the scientific aims of the program. The BEAT-HIV leadership attends monthly CEG meetings and shares information about research. For example, BEAT-HIV investigators have presented HIV cure-directed trial protocols to CAB and CEG members as they were being developed to seek input on trial design and participant-facing materials. BEAT-HIV scientists have also participated in community events, and have invited CAB members to present their perspectives in meetings organized by Collaboratory scientists.

In 2019, the BEAT-HIV leadership added a social sciences and ethics working group as an innovation to the CEG model. The BEAT-HIV social sciences and ethics working group was established to better understand community perceptions around HIV cure-directed research, assess participant experiences, and examine ethical aspects of HIV cure-directed science. The BEAT-HIV social sciences and ethics working group adopts a multi-disciplinary approach by bridging clinical and biomedical research, social and behavioral sciences, ethics, and community and patient engagement [16, 17]. BEAT-HIV social sciences and ethics projects receive input from CEG, and are implemented collaboratively to ensure a triangulated perspective. The working group reports to the Collaboratory leadership and receives funding as a BEAT-HIV sub-award (1.75% of total BEAT-HIV award) to implement community-based participatory research (CBPR) projects [18, 19]. Co-design and co-creation of research efforts are important features of this working group, which helps ensure the relevance of projects to PWH, affected communities, and the wider field of HIV cure-directed research.

#### Examples of BEAT-HIV CEG working groups and projects

The functionality of the BEAT-HIV CEG model is best illustrated through examples of BEAT-HIV CEG working groups and projects. Highlighted BEAT-HIV CEG working groups and projects illustrate how the CEG component(s) were instrumental in leadership, design, and implementation. These examples are embedded as vignettes and include: 1) BEAT-HIV analytical treatment interruption (ATI) position paper (CEG project) (Vignette 1), 2) BEAT-HIV cure-directed research education video series (CEG project) (Vignette 2), 3) BEAT-HIV participant experience study (CAB-led project with CEG support) (Vignette 3), 4) BEAT-HIV home-based viral load acceptability study (scientist-led project with CEG support) (Vignette 4), and Awareness of HIV Cure-Directed Research among HIV Clinic Patients in Philadelphia (scientist-led project with CEG support) (Vignette 5). Within each vignette, we share lessons learned using the CEG model.

Key cross-cutting learnings from BEAT-HIV CEG working groups and projects have been embracing a fluid research agenda that addresses evolving needs of the community. We have learned to be more intentional about when and how community feedback is requested. We try to allow ample time for pilot testing research projects, and remain mindful of varying literacy levels and learning styles in the community. We also learned about the importance of acknowledging participants’ lived experiences in HIV cure-directed clinical trials, and of engaging community members during the entire lifecycle of a project. As illustrated in Vignettes 3 and 4, providing working definitions of important concepts and building capacity in research methods is a necessity, together with making data available in understandable formats for non-scientific communities of interest, and engaging community members in dissemination activities.

#### Vignette 1: BEAT-HIV ATI Position Paper

(https://beat-hiv.org/wp-content/uploads/2016/09/BEAT-HIV-Position-Paper-FINAL.pdf) 
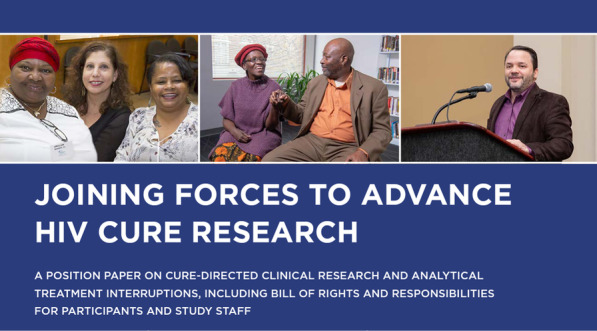


A key feature of HIV cure trials is the need to interrupt HIV treatment to determine the efficacy of interventions aimed at keeping HIV controlled in the absence of ART (60). Therefore, analytical treatment interruptions (ATIs) require community input to help ensure they remain ethical and acceptable to PWH and affected communities. In the first cycle of the BEAT-HIV Collaboratory (2016–2021), the CEG engaged its members in the creation of a position paper around ATIs in HIV cure-directed clinical trials.

Starting in 2017, CEG members identified and consolidated possible topics for inclusion and prioritization in the ATI position paper. From 48 possible topics identified, the CEG prioritized and consolidated five topics for inclusion, which were translated into the five modules of the ATI position paper. Module 1 focused on explaining what ATIs are and why they are needed in HIV cure-directed trials. Module 2 provided considerations for participation in HIV cure-directed trials. Module 3 outlined steps to navigate the informed consent process. Module 4 described social implications to consider when enrolling in ATI trials. Module 5 advanced considerations for women’s participation in HIV cure-directed research. In addition, the ATI position paper provided a Participant’s Bill of Rights and Responsibilities adapted to HIV cure-directed research, as well as community-friendly appendices such as a glossary of useful terms and additional resources.

A critical lesson learned from the ATI position paper experience has been the need to allow due process in prioritization of key topics surrounding ATIs and ample time to develop the modules to allow for adequate community consultation and input. The ATI position paper also served as an avenue for dialogue, capacity building and mutual understanding around ATIs in HIV cure-directed trials. The ATI position paper highlighted the need to acknowledge the participants’ lived experiences in HIV cure-directed clinical trials. As a result, vignettes featuring personal stories of individuals who have participated in ATIs were added. The ATI position paper further underscored the need to adopt community-friendly language when explaining complex HIV cure-directed research. While the BEAT-HIV leadership initially called the project a “White Paper,” the expression “ATI Position Paper” was eventually adopted after discussions with CAB members to avoid racial connotations. The ATI position paper was published online in 2020.

#### Vignette 2: BEAT-HIV Cure-Directed Research Education Video Series (The Philadelphia Story)

(https://beat-hiv.org/hiv-cure-education-series/)
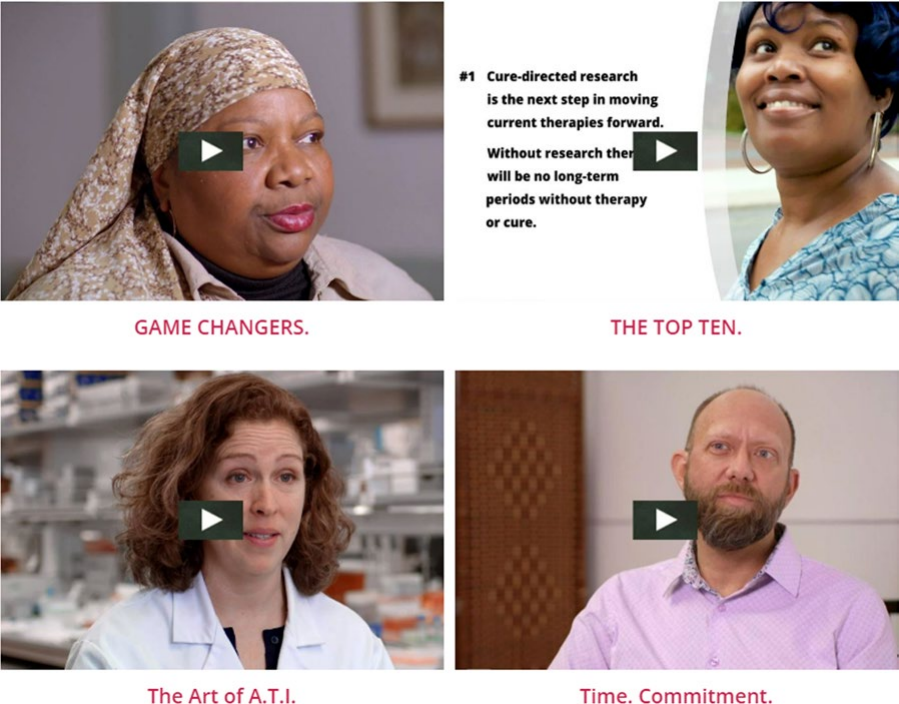


In the first cycle of the BEAT-HIV Collaboratory (2016–2021), the CEG engaged in the creation of an HIV cure-directed research educational video series titled The Philadelphia Story. The video series presented essential information by and to community members about participation in HIV cure-directed research, with special attention given to ATI trials. Videos document the perspectives of PWH, trial participants, HIV clinicians, and researchers about the possibilities, challenges, and ethics of HIV cure-directed research.

In 2019, this community education tool was collaboratively developed with different CEG constituencies with the aims of 1) translating complex clinical research into accessible language, 2) informing relevant stakeholders about research occurring in Philadelphia, and 3) explaining the importance of a robust informed consent process. CEG members were invited to participate in the video series at various stages of creation and assembly, including brainstorming and selecting topics of interest, developing a request for proposal (RFP) sent to Philadelphia-based video companies, selecting the production team via group deliberation, appearing as video participants, reviewing video footage, and critiquing video drafts. Philadelphia FIGHT hosted two community meetings to introduce the project, solicited feedback, and engaged community members in dialogue through the video series creation. Overall, 65 hours of film were reduced into four videos of 3–10 minutes each. The video series was premiered with a red-carpet celebration in June 2019 at End AIDS: The HIV Prevention and Outreach Summit, organized by Philadelphia FIGHT.

##### The four videos include the following:

1. Game Changers. Describes who and what is behind an HIV cure-directed study. Community, providers, case managers, and researchers come together to explain what to expect in a clinical trial.

2. The Top Ten. Reviews the top 10 items people should be aware of (and ask about) if considering joining an HIV cure-directed study.

3. The Art of ATI. Discusses what an ATI is and why it is included in HIV cure-directed studies.

4. Time. Commitment. Researchers and individuals who have participated in recent studies discuss what it takes to complete an HIV cure-directed clinical trial.

Video series organizers were mindful to ensure inclusivity in representation of video participants with respect to age, sex and gender, and race and ethnicity, with balanced representation between community and scientists’ perspectives.

#### Vignette 3: BEAT-HIV Participant Experience Study

The BEAT-HIV participant experience study was initiated under the first cycle of the BEAT-HIV Collaboratory (2016–2021) and continued into the second cycle (2021–2023). Because ATI studies present potential physical and psychosocial risks to trial participants (60,61), as well as risk of HIV transmission to partners (62), CAB members became interested in understanding the experience of study participants. CAB members stated that incorporating lived experiences would respect participants, honor the long history of community involvement in the advancement of HIV research, and adhere to the ethical principle of respect for communities in clinical research (63).
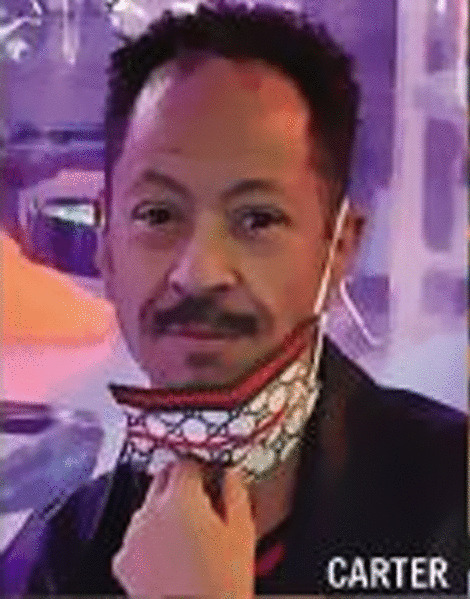


Starting in 2019, the BEAT-HIV participant experience study incorporated significant CAB member involvement in the design of the study, including the development of interview questions and analysis of the findings. The participant experience study was a nested sub-study within the BEAT-HIV trial “*A Pilot Phase I Randomized Study to Evaluate Innate Immune Activation Predictors of Sustained Viral Control in Adults with HIV Undergoing a Brief Analytical Treatment Interruption after Administration of Pegylated Interferon Alpha 2b in Combination with Two Intravenous Broadly HIV-1 Neutralizing Antibodies 3BNC117 and 10-1074*” (NCT03588715). The research design selected by the CAB included interviewing participants at two timepoints: 1) after enrollment into the trial (64), and 2) after participants had resumed their ART following the ATI. Various CAB and CEG members participated in meetings to discuss study findings and generate a set of considerations that were shared with BEAT-HIV clinical/biomedical scientists to inform future trials.

The BEAT-HIV participant experience study was informed by principles of community-based participatory research (CBPR), and CAB member engagement occurred throughout the entire research process including conceptualization, design, implementation, analysis, and communication of findings (18). The aims were to: 1) gain a richer understanding of participants’ experiences in HIV cure-directed clinical trials, 2) harness community expertise in the development of data collection tools and methods, as well as the analysis and interpretation of results, 3) develop hypotheses for future studies, and 4) inform scientists with considerations for best practices in the conduct of HIV cure-directed clinical trials.

#### Vignette 4: BEAT-HIV Home-Based Viral Load Acceptability Study

The BEAT-HIV home-based viral load acceptability study was initiated under the first cycle of the BEAT-HIV Collaboratory (2016–2021) and continued into the second cycle (2021–2023). ATI trials require participants to adhere to frequent monitoring visits for viral load tests. These visits can become burdensome to participants. Therefore, community members have advocated for ways to lessen the burden of the visit schedule. Tasso, Inc. and Merck & Co, Inc., Kenilworth, NJ, USA, developed an experimental home-based blood collection device that could facilitate on-demand viral load testing during ATI trials. In 2020, at the start of the COVID-19 pandemic, the BEAT-HIV CEG leadership decided to test the device as part of an ongoing HIV cure-directed ATI trial. In addition to including a reliability component (i.e., measuring whether the blood collected from the device could accurately measure viral loads), the BEAT-HIV CEG integrated a socio-behavioral research component focused on assessing acceptability. The home-based viral load acceptability study included three components: 1) nested in-depth interviews with participants testing the device as part of the ongoing BEAT-HIV ATI trial (65), 2) community-based in-depth interviews and focus groups to understand hypothetical acceptability of the device among PWH who were not enrolled in the study (66), and 3) community-based surveys to understand hypothetical acceptability of the device among diverse stakeholder groups, such as PWH, biomedical researchers, and HIV clinicians (67).
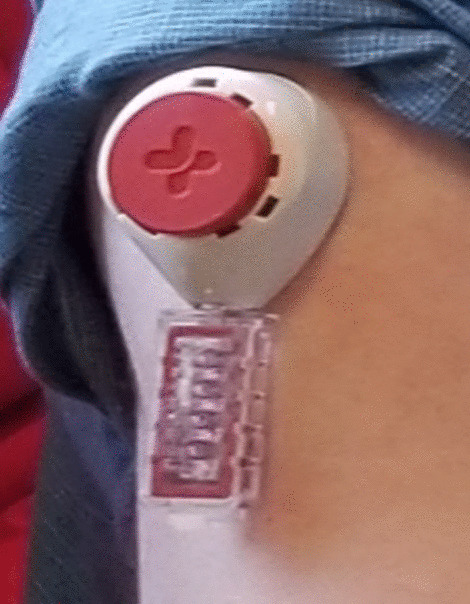


This effort involved the engagement of community members at all stages of the research process. BEAT-HIV CAB members provided input on research design and study instruments, helped moderate focus group discussions, and contributed to interpretation of findings and dissemination of results to the community as part of Philadelphia FIGHT’s HIV Prevention Summit in June 2022. Two BEAT-HIV CAB members participated in the ATI trial and generously contributed their lived experiences. In addition, BEAT-HIV CAB members helped review infographics and one-page lay-friendly summaries of study findings. The BEAT-HIV CEG also incorporated input on study design and instruments from four external community members from the AIDS Treatment Activists Coalition (ATAC) (a group of advocates advising on industry-sponsored research).

A critical lesson learned from the BEAT-HIV home-based viral load acceptability study included the importance of engaging community members during the entire lifecycle of the project (68)—as advisers, co-designers, participants, moderators, co-authors, and disseminators of research findings.

The BEAT-HIV CEG realized the benefit of having community members trained in research ethics and focus group moderation. Another lesson learned was the need to negotiate timelines for an industry-sponsored and milestone-based project, while finding a balance between seeking community input and moving the project forward at an acceptable pace. In our opinion, the inclusion of the socio-behavioral acceptability component into the main research protocol and the broad representation of CAB members as co-designers of research represent best practices for the field of HIV cure-directed research.

#### Vignette 5: Awareness of HIV Cure-Directed Research among HIV Clinic Patients in Philadelphia

During 2019, the third year of funding for the BEAT HIV Collaboratory v1.0, the CAB led the CEG in developing and implementing a survey of patients in HIV care in Philadelphia. The survey intended to assess the knowledge of and support for HIV cure-directed research to help develop and inform future community education and engagement projects. Understanding the awareness, perceptions, misperceptions, and interest in HIV cure research was seen as necessary to design meaningful content and effective delivery strategies for educational messages aimed at potential participants and the larger community of PWH. Information gained would address the limited data available to help guide the implementation of community education efforts for this population. 
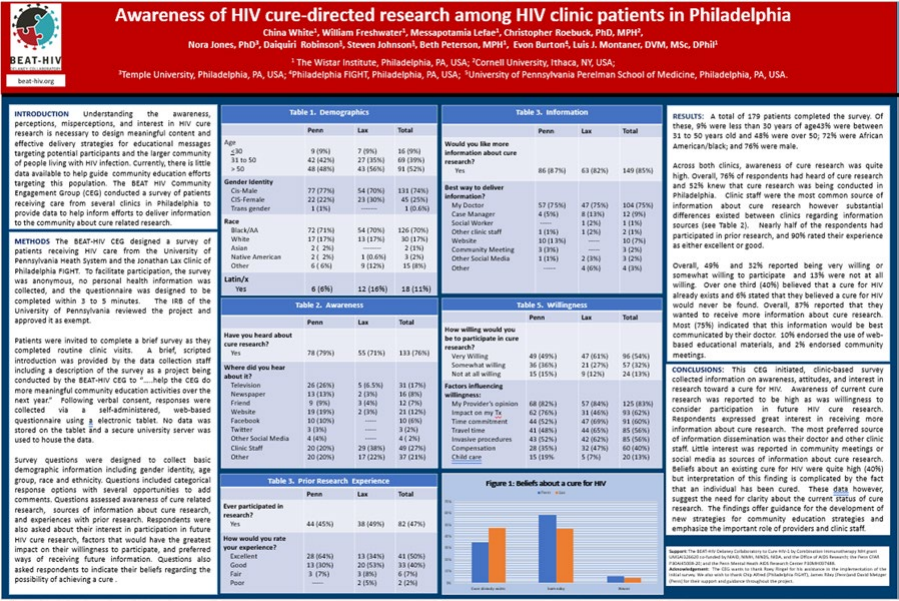


The survey was conducted among 177 patients receiving HIV care from the University of Pennsylvania Heath System and the Jonathan Lax Clinic of Philadelphia FIGHT. To facilitate participation, the survey was anonymous, no personal health information was collected, and the questionnaire was designed to be completed within 3 to 5 minutes while they completed routine clinic visits. Questions included categorical response options with several opportunities to add comments. Questions assessed the awareness of HIV cure-directed research, sources of information about cure research, and prior experiences with research. Respondents were also asked about their interest in participation in future HIV cure research, factors that would have the greatest impact on their willingness to participate, preferred ways of receiving future information, their beliefs regarding the possibility of achieving a cure for HIV.

This CEG project provided an opportunity for CAB members, investigators, and our community partner, Philadelphia FIGHT, to work together on a common project from conceptualization to presentation. Discussions of the purpose of the survey helped to crystalize the mission of the CEG. This process helped to highlight people in treatment for HIV as a priority population for the BEAT-HIV community engagement efforts. The selection of questions used in the survey was led by the CAB. This project also helped to address some of the power differentials inherent in the CEG framework and demonstrated equity among its three component groups.

### Lessons learned from BEAT-HIV CEG model

#### The CEG model

By recognizing the complementary role of the three CEG components, our community engagement model recognizes a common team. The CEG model advances a new model of community engagement beyond the traditional academic-community relationship centered on a CAB. Table 3 summarizes potential strengths and challenges of the CEG model that together constitute our collective reflective lessons learned from the BEAT-HIV CEG model to date.

**Table 3:** Lessons learned: potential strengths and challenges of the BEAT-HIV CEG model

Potential strengths
Creates synergy between goals of community-based organization, goals of community-advisory board, and aims of HIV research
Creates greater awareness of how to work together and creation of shared space
Overcomes shortcomings of traditional CAB-scientists model
Rectifies unequal power relationships that have been historical issues for CABs
Provides joint priority areas for collective input
Keep individuals engaged while maintaining meeting time to an acceptable level, and helps with multi-directional skill building for CEG members
Potential challenges
Independence of the CAB would be co-opted by direct linkages to investigators and CBO
CAB members may not be equally recognized for time commitments compared to other CEG members
Defining projects with community leadership (CAB- or CBO-led) versus input for basic or clinical sciences research
Ensuring community recommendations are incorporated into HIV cure-directed clinical trial design
Establishing clear yet iterative rules of engagement (e.g., commitment to building mutual understanding, respect, inclusion, equity, diversity and collaboration, among others)
Periodically define and refine roles and resonsibilities of CEG members, and to ensure equity in parties responsible for leading each project or working groups
Managing expectations regarding differential time commitments, availabilities and skills of CEG members
Avoiding conflating community engagement with recruitment efforts for clinical trials and participation in research

### Possible strengths of the CEG model:

#### Strength #1

The experiential, cultural, linguistic, and social expertise of community members is combined with contributions from basic, clinical/biomedical and social scientists, as well as ethicists in support of the BEAT-HIV Collaboratory’s multi-disciplinary HIV cure-directed research agenda. A key strength of the CEG model has been the synergy between the goals of the CBO (community engagement and implementation arm), the goals of CAB (sounding board and education/dissemination design) and the aims of HIV research (finding a safe, efficacious, acceptable, and scalable cure). Therefore, the CEG model moves its individual components beyond single or siloed missions and orients all groups toward a more equitable and holistic vision.

#### Strength #2

Greater awareness of how to work together. The CEG enables a shared and safe space while fostering greater self-awareness of individual members. Exchanges between the different CEG components help create unity across various experiences and facilitate empathy and mutual understanding. The CEG model is intentional in stressing that the whole become greater than the sum of its parts. The CEG model has the potential to generate more effective and community-relevant research emerging from robust partnerships and shared ethical commitments between the CBO, CAB, and scientists. As an overarching entity, the CEG acts as a dynamic bridge between different types of stakeholders. Its success, however, rests on the sustained involvement of all key players. Philadelphia FIGHT (CBO), together with the CAB, help ensure the community relevance of the research while facilitating design and implementation of projects. The CBO and CAB are attuned to community needs and establish an ongoing presence with respective affected communities.

#### Strength #3

The inclusion of the CBO helps overcome some of the shortcomings of the more traditional CAB-scientists model, which have been documented in the literature [13, 20–23]. For example, it can take years before research institutions develop trustworthiness with affected communities [24]. Inclusion of a CBO with deep-rooted relationships with the community moves engagement into a more productive and meaningful, rather than tokenistic, space [24, 25].

The CEG model incorporates a racial and social justice model into HIV cure-directed research and allows for Black and other communities to counter mistrust in the health care system and clinical research [26–28] due to historical ethical violations [29, 30]. This is important as racial and ethnic minority groups are less likely to report trust in their HIV care providers, which results in lower adherence to HIV treatment [31]. Lower rates of viral suppression may have downstream impacts for HIV cure research outcomes. We believe the CEG model may help build trust with the local community by using the CBO as a trusted messenger.

#### Strength #4

The three-way partnership helps to rectify unequal power relationships that have been historical issues for some CABs by creating a larger CEG team where each component is recognized as an active participant [23]. Importantly, the maintenance of the CEG model requires a willingness to engage in honest and candid conversations around meaningful community engagement in HIV cure-directed research. In a sense, Tuckman’s stages of team development (i.e., forming, storming, norming and performing) [32] became an ongoing reality and an iterative process. The CEG model has allowed the BEAT-HIV Collaboratory to more effectively advance principles of community empowerment by creating a larger community team where each group is an equal partner in support of the other two. The interdependence created by this structure reinforces equity and mutual respect under a shared vision to advance research toward an HIV cure.

#### Strength #5

Based on the limited guidance in the literature on best practices for participatory approaches in HIV cure-directed research, the CEG structure organically emerged as the joining of three critical groups required to effectively deliberate, plan and implement HIV cure-directed clinical trials helping to identify joint priority areas for collective input. For example, the BEAT-HIV ATI position paper (highlighted in Vignette 1) provides community-focused participatory guidelines and a bill of rights and responsibilities for both participants and investigators. The BEAT-HIV position paper highlights the impact of all CEG members acting together, as none could have generated an authoritative position without the others.

#### Strength #6

To maintain the amount of meeting time each month to a manageable level, regular CAB meetings are held within the model of CEG meetings with CAB leadership (i.e., Chair, Secretary) who also serve comparable functions in the CEG. Meeting agendas address both CEG and CAB agenda items. In the event a vote is needed on a CAB-only item (i.e., CAB operating procedures), only CAB members vote, whereas all members vote on CEG items.

While BEAT-HIV CAB leadership has evolved over the years, we recognized the need to have everyone be engaged and included as much as possible regardless of roles. The BEAT-HIV CEG experience has shown the willingness to invest in knowledge transfer and skill building for BEAT-HIV CAB members on an ongoing basis, such as leadership, public speaking, or writing skills. The BEAT-HIV CAB decided to create an orientation packet for new members to reduce the gap in information between new and longer-serving community members regarding the role of a CAB and CEG. The BEAT-HIV CEG prioritized capacity building that was multi-directional, including scientists learning new skills in community literacy and cultural awareness as well as community members learning more about HIV cure-directed science.

### Possible challenges of the CEG model

#### Challenge #1

One of the first obstacles encountered with the CEG model was the fear that the independence of the CAB would be co-opted by direct linkages to the investigators and CBO. We addressed this by ensuring that each component group within the CEG recognized the independence and value of the other component groups in achieving the common goal. The CEG is a community engagement collaborative and the power differentials between the component parts needed to be acknowledged and closely monitored. For example, although CAB meetings occur in the presence of CEG members, the CEG Chair (who is also the CAB Chair) has the option to extend a CAB meeting by going into a CAB member-only portion at end of agenda if there is an item the CAB wants to discuss outside of the larger CEG group.

#### Challenge #2

Research efforts toward an HIV cure have not witnessed the same passionate urgency as the life-saving HIV treatment research of the 1980s and early 1990s [33], and rely largely on the altruism of otherwise healthy volunteers to participate in research and as community representatives [34–36]. While CAB members serve as volunteers, the CBO and scientists serve while supported by institutional salary effort. The increasing time commitments required to execute CEG projects could result in CAB members not being equally recognized or compensated for their time commitment. We found it remained important to seek strategies to recognize the added time commitments beyond group meetings that CAB partners are requested to contribute [28, 29]. For example, food is provided at meetings, festive seasonal “thank you” parties are held (e.g., winter season), recognition of a CAB member of the month is included in monthly meetings, provision of meeting travel support for poster presenters from the CAB, provision of iPads to CAB members in need of support for online attendance, and additional stipends for CAB participation in CEG-related planning meetings or sub-committees are provided to account for time spent beyond standing CAB meetings.

#### Challenge #3

Although essentially a basic sciences effort (involving human cells, mechanistic pathways, genes, and animal models), HIV cure-directed research has important significance for a broad range of constituency groups across the translational research continuum [37]. For PWH, the hope of a cure has significance around perceptions of health and potential for research participation. For HIV clinicians, HIV cure-directed research could help improve clinical and psychosocial outcomes for PWH. While a cure for HIV infection is an aspiration for the future, advancing cure-directed efforts still need PWH to be willing to enroll in observational studies, interrupt ART, and take up interventions. Defining community input and benefit for basic sciences research is important, as it is unlikely that participants will derive direct clinical benefits from the research in the near future [38, 39]. In response to this challenge, we created the BEAT-HIV social sciences and ethics working group to provide a bridge between basic and clinical sciences, and community priorities. BEAT-HIV CAB members can have more direct input into social sciences research than basic and translational sciences research.

#### Challenge #4

HIV cure-directed research efforts remain in the early stages, and a key challenge has been to ensure that community recommendations are incorporated into HIV cure-directed clinical trial designs. Study protocols are often almost fully developed before they are discussed with community members given funding and iterative research project cycle constraints. Pantelic and colleagues coined the expression “epistemic injustice” to describe circumstances when community input was solicited, but not acknowledged or applied [11].

While the NIH permitted clinical trials to occur in the first iteration of the BEAT-HIV Collaboratory, the NIH did not allow clinical trials in the second iteration of the Collaboratory. As a result, BEAT-HIV Collaboratory scientists proposed a clinical trial to the AIDS Clinical Trials Group (ACTG), which received subsequent approval for development. A BEAT-HIV community representative is included as a member of the ACTG protocol team to ensure community feedback is incorporated into the research.

#### Challenge #5

The CEG model necessitated the establishment of clear yet iterative rules of engagement within the group's by-laws (provided in orientation packets for all new CEG members) to avoid unintended conflict due to inherent group dynamics by diverse membership with differences in life experience (persons with primary experience with poverty, violence, etc.), power dynamics (gender, racial, educational, financial), expectations of how the group should function, and concerns on confidentiality for those electing to keep their HIV status private. By reading the mission of the group at the start of each CEG meeting, we also focus the group on building mutual understanding, respect and seeking common ground towards a unified goal. Prospective CAB members are also required to attend three meetings before they can be voted in. This period orients them in advance as to how they should engage as a member of the group. Additional examples where rules of engagement have been useful include defining how the public disclosure of reports or publications from the CEG would be approved, obtaining confidentiality agreements from all members, obtaining consent for any pictures of CAB members to be shown publicly, etc. However, at times, it became necessary to engage in periods of re-adjustment and pursue resolution of conflict by smaller subgroups or by calling in BEAT-HIV leadership or outside trusted community mediators to reinforce our CEG mission.

#### Challenge #6

Another major lesson learned has been the need to periodically define or refine roles and responsibilities, and identify clear responsibilities for the party/parties leading projects or working groups, as illustrated in Vignettes 1–5. The “ownership” of each project or working group became more important to define over time. These roles and responsibilities enabled clarifying expectations and levels of participation. To facilitate these delineations, we adopted a five-fold taxonomy of 1) informing, 2) consulting with, 3) involving, 4) partnering with, and 5) having the CEG lead to describe how a project would be performed. By defining whether a project would be CEG-, CBO-, CAB- or scientist-led before assigning collaborative roles to all, we could ensure that expectations and roles were clear. Over time, intentionally defining the roles that each group had while acknowledging leadership opportunities available to all resulted in greater community member appreciation for the time and commitment of CEG members.

#### Challenge #7

Because most CAB members have external work commitments outside of the CAB, while CBO members and scientists usually perform CEG activities as part of their professional role, we have needed to manage expectations regarding differential time commitments, availabilities, and skills. It has been essential to monitor not overburdening CAB members with tasks and sub-committee assignments. The adage “less is more” became useful to ensure issues could be properly championed at defined times. As suggested by Wilkinson and colleagues, we found that engagement should be tailored and “dosed” appropriately, based on the intensity of the research and sensitivity of the topic [40]. To mitigate this challenge, we reminded BEAT-HIV CEG members to be intentional about honoring differing preferences, interests, motivations, and incentives of CEG members.

#### Challenge #8

It is important to avoid conflating community engagement with recruitment efforts for clinical trials and participation in research [3, 41]. The CEG model allowed investigators to present clinical trials open for recruitment to community partners. However, it was important to note that information was being provided for dissemination and awareness rather than expecting that CEG members were “recruiters” or accountable for success in enrollment. Review of enrollment totals with specific mandates to support any one study presented is not allowed. This guideline has been well received by clinical investigators.

### Advancing the field of community engagement in HIV cure-directed research

By embracing an intentional approach to community engagement and viewing community members as subject matter experts, we hoped to challenge traditional notions of maintaining isolated pockets of “expertise,” whether scientific or community [42]. As such, the CEG model helps move us toward a more integrated and equitable vision of HIV cure-directed research and contributes to building long-term trust and goodwill investments with affected communities. The historical relationship with Philadelphia FIGHT as a CBO partner has been a key factor upon which to build the expanded CEG model.

Despite the 2013 NIH mandate to include community engagement in MDC-funded HIV cure-directed research efforts, there has been limited documentation of successful models and best practices around meaningful engagement. In Australia, Lau and colleagues shared learnings from community engagement around a hypothetical HIV cure-directed trial, in which approximately 40 individuals from different sectors (e.g., basic scientists, HIV clinicians, PWH, community members, social scientists, bioethicists) engaged in role play exercises to improve education and communication between stakeholder groups [41]. This experiment underscored the need for a critical approach to inclusion, since not all groups have equitable capacity to meaningfully participate [41]. Our CEG model followed this principle by stressing equitable inclusion in its structure [3].

Notwithstanding the lack of a published knowledge base, notable lessons can be learned from HIV treatment and prevention research. For example, Lo and colleagues reviewed historical examples of stakeholder engagement in HIV clinical research [43]. HIV treatment advocacy and engagement was robust from the onset and led to revolutionary therapeutic advancements for PWH in the late 1980s and early 1990s [43]. In contrast, the late community engagement in early PrEP research backfired and delayed understanding of downstream PrEP implementation challenges [41]. Experience from the field of preventive HIV vaccine research showed that early and sustained engagement could be critical in paving the way for sequential trials [43]. Brocher and colleagues compares standardized metrics to evaluate recruitment practices in two HIV monoclonal Antibody Mediated (AMP) clinical trials, and found successful strategies varied between regions, underscoring the need to tailor strategies to local contexts [44].

The field of HIV cure-directed research rests on four decades of community-engaged approaches to propel HIV-related discoveries forward. The field of HIV prevention research is particularly full of examples of community engagement—whether they were successful or not [4, 40, 45–49]. For example, a fundamental lesson from HIV prevention trials has been the contribution of the socio-behavioral sciences in providing an empirical foundation for community-engaged research, and knowledge translation to support the long-term process of scientific discoveries [46]. In examining facilitators and challenges to meaningful community engagement in four countries, Newman and colleagues identified the following cross-cutting themes as essential: increasing research literacy (including addressing misconceptions), acknowledging mistrust in biomedical research due to historical exploitation, engaging early and often with participatory processes, and developing appropriate stakeholder roles. These considerations inspired us as we continue to implement community engagement activities, and as we evolve the BEAT-HIV CEG framework with deliberate integration of socio-behavioral sciences and ethics research.

### Unanswered community engagement questions and looking into the future

Seven years into the formation of the BEAT-HIV CEG model, we still have several unanswered questions. One conundrum we continue to face is the need to create benefits for the community beyond the long-term goals of HIV cure-directed research. This creation of community benefits aligns with the ethical principles of beneficence, justice, and social value of research. The question remains how to create meaningful community benefits when funding mechanisms have strict research mandates as defined by requests for proposal. For example, we wonder if it would be possible to establish a certification or training program for CAB members so they could receive external recognition for their service and build transferable skills beyond HIV cure-directed research. The strengths and challenges of the BEAT-HIV CEG provide support for ongoing advocacy efforts geared toward changing funding restrictions.

Furthermore, as we continue to engage in CBPR, lines can become blurred between “researchers” and the “researched” [50], or between scientists and community. We recognize that engagement of community members as co-researchers can translate into higher expectations and demands on them [42]. The value of engaging additional CBOs as partners in the CEG model is another question for future development, as research teams often may cover diverse geographical areas not otherwise addressed by a single CBO.

In addition to better defining GPP around community engagement in HIV cure-directed research, the field could significantly benefit from establishing monitoring and evaluation standards to define acceptable community engagement. The paucity of the community engagement literature in HIV cure-directed research, contrasted with rapid growth of investments in basic and clinical/biomedical research, highlights the urgency of proposing best practices for meaningful engagement. MacQueen and colleagues [51] called for dedicated efforts aimed at evaluating community engagement in terms of processes, outcomes, and longer-term impacts. In a similar effort, Staley and colleagues identified nine categories of community engagement impacts, including effects on the research agenda, research design and delivery, research ethics, people involved, researchers, participants, the wider community, community organizations, and overall implementation and change [52]. In the context of comparative effectiveness research, Edwards and colleagues proposed a 10-step continuum framework to guide researchers in selecting appropriate approaches for meaningful patient engagement [53]. These steps include: 1) soliciting topics, 2) prioritizing research, 3) framing questions, 4) selecting outcomes, 5) creating conceptual framework, 6) analyzing the plan, 7) collecting data, 8) reviewing and interpreting results, 9) translating results, and 10) disseminating findings [53]. The National Academies of Sciences, Engineering and Medicine (NASEM) recently proposed a conceptual model for assessing meaningful community engagement with a focus on health equity through transformed health systems [54]. They stated that “the fundamental question is not whether entities think they are engaging communities but whether communities feel engaged [54].” Health equity values of diversity, inclusivity, partnerships, opportunities, acknowledgement, visibility, sustained relationships, mutual respect, trust, shared power, and structural support for community engagement, should become key goals of meaningful engagement and should be added to key definitions of community engagement and PPI. Documenting lessons learned from the continued development of the CEG model, and establishing transparent guidance to evaluate the impact of community engagement efforts, could help increase accountability and equity between community stakeholders and scientists.

### Limitations

Our work has several limitations. We provide a single case study of our experiences, and did not conduct a formal external evaluation of the BEAT-HIV CEG model, which would have likely yielded a more objective account. Assessment of BEAT-HIV CBO and CAB members’ perspectives about the CEG model would require separate empirical research. The CEG model could benefit from more formal evaluation of its process and productivity in the future. Further, our group does not have the authority to prescribe guidance, metrics, and outcomes on meaningful community engagement in HIV cure-directed research, as this would require broad stakeholder input. Our CEG model will continue to evolve, and we hope to incorporate new guidelines on community engagement in HIV cure-directed research as they become available. The manuscript was drafted by a social scientist (first author), with input from BEAT-HIV-affiliated CAB and CEG members. While the social scientist is not physically based in Philadelphia, she interfaces with the BEAT-HIV community and CEG members on a twice-monthly basis via teleconference. Further, a potentially untapped opportunity would be to integrate community engagement lessons learned from other fields, such as CBPR [19, 55], organizational behavior [56], COVID-19 [57, 58] or oncology research [59], among others.

## Conclusions

Heightening the centrality of community-engaged approaches will yield dividends in the future ethical conduct and implementation of HIV cure-directed research, and the eventual roll-out of an HIV cure. We present a novel approach to community engagement in HIV cure-directed research. We believe our community engagement group model integrating a CBO, CAB and scientists allowed for a more effective, equitable and ethical approach of engagement in our community. In sharing our lessons learned and challenges, we contribute to the science of community engagement in HIV cure-directed research. Our model illustrates the importance of partnerships and establishing clear expectations between constituent groups. The CEG is an outgrowth of the more traditional CAB-only model for community engagement, reflecting a growing trend to integrate community into working teams rather than limited to working as independent advisory groups. Importantly, the CEG model does not replace CAB-only roles, but rather provides a team-based model for joint action. As research funders aim to include community input into basic, clinical/biomedical, social and ethics research, we believe that the benefits of the CEG model may allow for funding resources able to support leadership and participation by all three components. Above all, we hope our experiences with the CEG model will advance discussion on how to engage communities more meaningfully, ethically, and sustainably in research.

## Supplementary Information

**Additional file 1**. **Appendix I:** Generalized CAB/CEG Agenda Example.

## Data Availability

Data sharing is not applicable to this article as no datasets were generated or analyzed as part of this manuscript.

## Abbreviations

ACTG: AIDS Clinical Trials Group
AMP: Antibody Mediated Prevention
ART: Antiretroviral Treatment
ATAC: AIDS Treatment Activists Coalition
ATI: Analytical Treatment Interruption
AVAC: [Formerly] AIDS Vaccine Advocacy Coalition
BEAT-HIV: Beyond Antiretroviral Therapy – HIV
CAB: Community Advisory Board
CBO: Community-Based Organization
CBPR: Community-Based Participatory Research
CDC: U.S. Centers for Disease Control and Prevention
CEG: Community Engagement Group
DEIA: Diversity, Equity, Inclusion and Accessibility
FQHC: Federally Qualified Health Center
GIPA: Greater Involvement of People with HIV
GPP: Good Participatory Practice
MDC: Martin Delaney Collaboratory
NASEM: National Academies of Science, Engineering and Medicine
NGO: Non-Governmental Organization
NIAID: National Institute of Allergy and Infectious Diseases
NIH: National Institute of Health
PPI: Patient and Public Involvement
PrEP: Pre-Exposure Prophylaxis
PWH: People with HIV
RFP: Request for Proposal
UNAIDS: United Nations Programme on HIV/AIDS
WHO: World Health Organization
