# Supplementary material for: Community engagement group model in basic and biomedical research: lessons learned from the BEAT-HIV Delaney Collaboratory towards an HIV-1 cure
Source: Res Involv Engagem. 2023 Jun 8;9:39. doi: 10.1186/s40900-023-00449-y (PMC10248979; doi:10.1186/s40900-023-00449-y)
Supplement: Supplementary file 1 — Additional file 1. Appendix I: Generalized CAB/CEG Agenda Example. [file 40900_2023_449_MOESM1_ESM.docx]

GENERALIZED CAB/CEG AGENDA EXAMPLE

 **BEAT2.0 HIV**

**Community Advisory Board/CEG Meeting**

**Date**

**Time**

**In-Person:** Address

**Zoom Meeting:** Connection details

*VISION STATEMENT*

*A world where HIV and AIDS research meaningfully involves impacted and affected communities, is collaboratively created, and is openly shared.*

*MISSION STATEMENT*

*As a Philadelphia based HIV Cure Research Community Advisory Board/CEG our Mission is to:*

- *Integrate community involvement in HIV and AIDS cure related research and clinical trials.*
- *Serve as a bridge community to provide input and feedback to BEAT HIV projects.*
- *Foster and maintain communication and partnerships with project researchers in order to promote transparency and to disseminate findings in HIV cure research to our communities.*

**AGENDA**

**Time** **Welcome** – (CAB/CEG Chair)

Reading of Vision & Mission Statement

**Time Roll Call** / **Attendance**: (CAB/CEG Secretary)

**Attendance:**

**CAB/CEG Officers & Members:**

Chair, **Co-Chair, Secretary**

**CAB Members: 8-14 members**

**CEG: CAB Faculty Advisor, BEAT-HIV MPIs, Philadelphia FIGHT Outreach Representative,** BEAT-HIV Program Administration, Social Scientists.

**CAB/CEG STAFF: Community engagement coordinator**

**Guests: 1-3 attending usually**

**Excused attendances**: listed

**Time** **CAB Projects**

- Task Tracker (CAB/CEG Co-Chair)

| **Task** | **Task Leader** | **Update** | **DEADLINE** |
| --- | --- | --- | --- |
| **Task 1** | **Person name** | Update notes |  |
| **Task 2** | **Person name** | Update notes |  |
| **Task 3** | **Person name** | Update notes |  |

- **Project 1** (Person leading effort)
- Available & Review
- **Project 2** (Person leading effort)
- Update
- **Project 3** (Person leading effort)
- Update

**6:30 CEG Projects**

- **Project 4** (Person leading effort)
- **Social Sciences and Ethics Working Group**
- **Chairperson’s Report (CAB Chair)**
  - New prospective CAB member voting
- **Meeting Attendance**
- **Development Committee Update**

**Time BEAT PI Report on Research efforts** (BEAT MPI attending)

**Time Co-Chairperson’s Report on upcoming initiatives** (CAB/CEG Co-Chair)

**Time Community Partner Update for FIGHT** (Philadelphia FIGHT Person leading effort)

**Time CAB member community participation monthly report-**

- *CAB members please report on any activity you were/will be engaged in this month that speaks to the mission of the BEAT HIV CAB.*

**Time Other Business**

- Upcoming events/opportunities for outreach

**Time Adjourn**
